# EXOSC5 as a Novel Prognostic Marker Promotes Proliferation of Colorectal Cancer via Activating the ERK and AKT Pathways

**DOI:** 10.3389/fonc.2019.00643

**Published:** 2019-07-18

**Authors:** Hongda Pan, Jingxin Pan, Shibo Song, Lei Ji, Hong Lv, Zhangru Yang

**Affiliations:** ^1^Department of Gastric Surgery, Fudan University Shanghai Cancer Center, Fudan University, Shanghai, China; ^2^Department of Hematology, The Second Affiliated Hospital of Fujian Medical University, Quanzhou, China; ^3^Department of Oncology, Shanghai Medical College, Fudan University, Shanghai, China; ^4^Department of Gastrointestinal Surgery, Beijing Hospital, Beijing, China

**Keywords:** EXOSC5, proliferation, colorectal cancer, Akt signaling pathway, ERK signaling pathway, prognosis

## Abstract

**Background and Objective:** Exosome component 5 (EXOSC5) is a novel cancer-related gene that is aberrantly expressed in various malignances. However, the molecular mechanism and biological role of EXOSC5 have not been explored in colorectal cancer (CRC). In this study, we investigated the functions and mechanisms by which EXOSC5 promotes the progression of CRC.

**Methods:** EXOSC5 expressions in CRC cell lines and paired CRC and adjacent normal tissues were measured via quantitative real-time PCR (qRT-PCR), Western blot and immunohistochemistry (IHC). *In vitro* experiments including colony formation, Cell Counting Kit-8 (CCK-8), and flow cytometry and *in vivo* tumorigenesis assay were performed to explore the effects of EXOSC5 on growth of CRC. The impacts of EXOSC5 on ERK and Akt signaling pathways were measured by Western blot.

**Results:** The mRNA and protein expression levels of EXOSC5 were up-regulated in CRC as compared to adjacent normal tissues. IHC analysis indicated that high EXOSC5 level was positively associated with poor prognosis. EXOSC5 overexpression facilitated the growth of CRC cells, while EXOSC5 knockdown led to decreased proliferation, G1/S phase transition arrest. The oncogenic functions of EXOSC5 were associated with activation of the ERK and Akt pathways in CRC.

**Conclusion:** EXOSC5 is overexpressed in CRC and promotes CRC growth partly through activation of ERK and Akt signaling pathways. Accordingly, EXOSC5 may be a novel oncogene, and acts as a therapeutic target, or prognostic factor for CRC.

## Introduction

Colorectal cancer is one of the major threats to public health; it is the third most common malignancy and the third-leading cause of cancer-related deaths globally (1). CRC is a heterogeneous malignancy with complex carcinogenic mechanisms. Although great efforts have been made to promote the management of CRC, the prognosis of CRC patients is far from satisfactory. Accordingly, it is important to explore the underlying mechanisms of CRC to promote the development of optimal therapeutic strategies and identification of promising diagnostic biomarkers.

The exosome complex is a multi-protein intracellular complex capable of degrading various types of RNA molecules. Apart from their function in RNAs degradation, components of the exosome complex have been associated in carcinogenesis. Goodarzi et al. indicated that transfer RNAs (tRNA) were promoters of breast cancer metastasis, in which tRNAGluUUC promoted metastasis by directly enhancing EXOSC2 expression (2). Bauer et al. found that EXOSC3 was significantly associated with survival, and was included into a gene expression-based risk score for treatment guidance in gastric cancer (3). Stefanska et al. demonstrated that depletion of EXOSC4 specifically and effectively suppressed cancer cell growth and cell invasion in various types of cancer, indicating an oncologic role for EXOSC4 in driving cancer development (4). EXOSC5 is a non-catalytic component of the RNA exosome complex. Guo et al. demonstrated that EXOSC5 was recruited by and directly interacted with the Zinc-finger antiviral protein to degrade the target RNA (5). Previous studies have reported that EXOSC5 was overexpressed in various epithelial and hematopoietic tumor and cell lines (6–8), however, the functional and prognostic roles of EXOSC5 in CRC carcinogenesis are largely unclear.

Our study gave evidence of a significant correlation between the up-regulation of EXOSC5 and survival outcomes of patients with CRC, and the oncogenic function was investigated via *in vivo* and *in vitro* experiments. Moreover, we have shown that EXOSC5 promoted CRC progression via ERK and Akt signaling pathways. As far as we know, this is the first study reporting the oncogenic and prognostic roles of EXOSC5 in the development of CRC.

## Materials and Methods

### Patients and Specimens

A total of 53 primary CRC tissues and paired normal tissues were collected from the Department of Gastrointestinal Surgery, Beijing Hospital (BJH). All tissues were frozen immediately in liquid nitrogen after surgical excision and stored at −80°C. From June 2011 to April 2013, archival formalin fixed and paraffin-embedded specimens of 159 patients with CRC who underwent colorectal surgery at BJH were obtained from the Department of Pathology. Written informed consent was obtained from all patients and the study was approved by the ethics committees of the BJH.

### Cell Lines and Cell Culture

The immortal normal colon cell lines (NCM460) and CRC cell lines (HT29, SW480, SW620, CACO2, and LOVO) were purchased from American Type Culture Collection (Manassas, VA, USA). Cell lines were cultured in Dulbecco's modified Eagle's medium (DMEM) (Gibco) with 10% fetal bovine serum (FBS). Dissolved by DMSO, AKT inhibitor MK-2206, or ERK inhibitor GDC-0994 (Selleck Chemicals, USA) were administered to suppress the phosphorylation of AKT or ERK signaling pathways. All cells were kept with 5% CO2 humidified atmosphere at 37°C.

### Lentivirus Constructs and Transfection

EXOSC5 short hairpin RNAs (shEXOSC5), EXOSC5 overexpression plasmid, and its control were purchased from GeneChem Company (Shanghai, China). The target sequences of the shRNAs were as follows: EXOSC5 shRNA#1: 5′- GAAGGTCAGCAAAGAGATT−3′; EXOSC5 shRNA#2: 5′- CGAAGTGATCCTGAGGCCGAAGATT−3′. HT29 and SW480 cells were transfected with the shEXOSC5 plasmid, and CACO2 and LOVO cells were transfected with the EXOSC5 overexpression plasmid. Cells transfected with vector were used as controls. The shRNAs and plasmids were transfected into cells using Lipofectamine 2,000 reagent (Invitrogen) following the manufacturer's protocol. Knockdown or overexpression of EXOSC5 was measured by qRT-PCR and western blot.

### RNA Extraction and qRT-PCR Assay

TRIzol reagent (Invitrogen, Carlsbad, CA, USA) was used to extract the total RNA from tumor and normal tissue samples and cultured cells. PrimeScript RT Reagent Kit (Takara, Shiga, Japan) was used to synthesize complementary DNA (cDNA). SYBR Green Premix Ex Taq (Takara, Shiga, Japan) on an ABI 7,900 PCR system (Applied Biosystems) was used to conduct qRT-PCR. The primer sequences used were: EXOSC5 forward: 5'- ACTTTGCCTGCGAACAGAACC−3′, EXOSC5 reverse: 5′- CTCTTTGCTGACCTTCACCTC−3′; GAPDH forward: 5′- TGACTTCAACAGCGACACCCA−3′, and GAPDH reverse: 5′- CACCCTGTTGCTGTAGCCAAA−3′. GAPDH was used as endogenous control. The relative expression level of the target gene was calculated by 2-ΔCT and normalized to control cells. For the correlation study, the fold change of EXOSC5 expression level was calculated by 2-ΔΔCT.

### Protein Extraction and Western Blot Analysis

The total proteins were extracted on ice for 60 min in RIPA buffer (Thermo Scientific, USA) with phosphatase inhibitors and protease (CST, USA). Cell lysates were centrifuged at 1.2 × 10^4^ g, 4°C for 20 min. The concentrations of the supernatants were detected using the BCA Protein assay kit (Thermo Scientific, USA). Identical quantities of proteins were electrophoresed by SDS-PAGE (Life Technology, USA), transferred onto PVDF membranes (Millipore, USA). After that, total proteins were incubated with primary antibodies at 4°C overnight. EXOSC5 was detected with a polyclonal antiEXOSC5 antibody (Abcam, USA). Then, the membrane was washed with PBS three times and incubated with secondary antibody. GAPDH (CST, USA) was used as standard loading control.

### Immunohistochemistry (IHC)

The IHC was conducted using a standard immunoperoxidase staining procedure. The primary antibodies against EXOSC5 (Abcam, UK) were used at concentrations of 1:200. The percentage of stained cells was scored as 0 (no staining), 1 (1–25%), 2 (26–50%), or 3 (51–100%). The staining intensity was scored as 0 (negative), 1 (weak), 2 (intermediate), or 3 (strong). The IHC score was calculated as the sum of both parameters, and the samples were grouped as negative (0), weak (1–2), moderate (3), and strong (4–6) staining. The tissues scored 0–3 were considered low expression, while those scored 4–6 were deemed high expression.

### Cell Proliferation and Colony-Formation Assays

The cell proliferation assays were performed with a CCK-8 Assay. At 48 h after transfection, cells were trypsinized and reseeded into 96-well plates (3,000 cells/well). Then, 10 μL of CCK-8 (Dojindo, Kumamoto, Japan) solution was added to each well and absorbance at 450 nm was measured after 2 h of incubation. Anchorage-independent growth was determined by colony formation assays. 500 cells were seeded in 6-well plates, and were cultured for 2 weeks. The cells were then fixed using 4% paraformaldehyde, and stained with 0.1% crystal violet (Sigma, St. Louis, MO). The number of colonies with more than 50 cells was counted. The experiments were repeated in triplicate.

### *In vivo* Tumorigenic Assays

Four to six weeks old nude mice were obtained from the Shanghai Laboratory Animal Center of the Chinese Academy of Sciences (Shanghai, China). The animal studies were approved by the Fudan University Animal Ethics Committee. All the animals were housed and maintained under specific pathogen-free conditions. Hundred microliter PBS with 1 × 10^6^ CRC cells were injected subcutaneously into the dorsal region of nude mice. Tumor volume (mm^3^) was measured every 5th day, and tumor weight (mg) was measured at the end of the experiment.

### Flow Cytometric Analysis of Cell Cycle

The cells were fixed with 75% ethanol at 4°C overnight. Cells were stained with 50 μg/mL propidium iodide (PI; Kaiji, China) containing RNaseI (Kaiji, China). The stained cells were analyzed by flow cytometry (Guava® easyCyte™). The experiments were performed in triplicate independently.

### Statistical Analysis

ANOVA test was performed to compare the differences between groups. Kaplan-Meier with the log-rank test were used to analyze Survival data. Cox proportional hazards regression model was used to identify independent prognostic factors associated with overall survival. The correlations between IHC scores and clinicopathologic characteristics were tested using chi-square test and Logistic multivariate analysis. A *P* < 0.05 was considered statistically significant. All statistical analyses were performed by SPSS version 21.0 (IBM, USA).

## Results

### EXOSC5 Expression in CRC Cell Lines and Tumor Tissues

**Figure 1:**
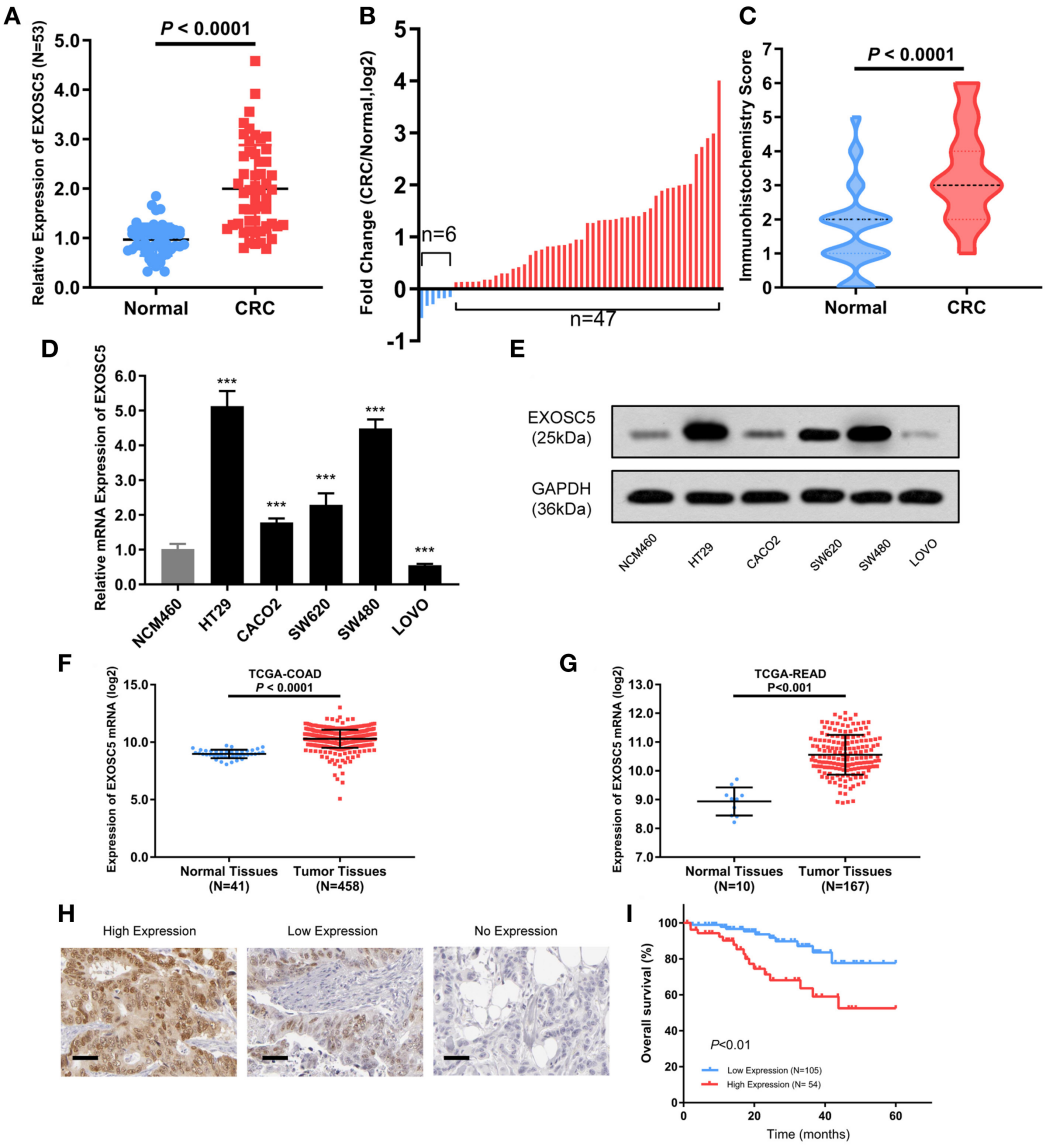
EXOSC5 was overexpressed in CRC tissues and cells, and correlated with overall survival. **(A,B)** EXOSC5 mRNA levels in 53 pairs of tumor samples and matched normal tissues were determined by qRT-PCR. **(C)** EXOSC5 protein expression in CRC and normal tissues was measure by IHC staining. **(D,E)** mRNA and Protein levels of EXOSC5 in human colon cell line and five CRC cell lines measured by qRT-PCR and Western blot, respectively. **(F,G)** EXOSC5 expression was increased in tumor tissues compared with normal colorectal tissues in TCGA-COAD and TCGA-READ datasets. **(H)** Representative IHC staining for EXOSC5 in CRC tissues (scale bar: 50 μm; magnification: 200X). **(I)** High EXOSC5 expression correlated with worse overall survival in CRC patients.

EXOSC5 mRNA expression levels were determined in 53 matched CRC and adjacent non-tumor tissue samples by qRT-PCR (Figure 1A). EXOSC5 expression level was significantly increased in 88.7% (47/53) of CRC tissues as compared to that in normal tissues (Figure 1B). The high expression of EXOSC5 protein in CRC was validated by immunohistochemistry (Figure 1C; Table S1).

Further, we detected expression levels of EXOSC5 mRNA and protein in CRC and colon cell lines by qRT-PCR and western blotting analysis. The results showed that EXOSC5 was significantly increased in HT29 and SW480 cells, and had relatively low expression in CACO2 and LOVO (Figures 1D,E).

In order to verify our findings, the gene expression of EXOSC5 was explored in The Cancer Genome Atlas (TCGA). The result revealed that EXOSC5 was remarkably up-regulated in Colon Adenocarcinoma (COAD) and Rectal Adenocarcinoma (READ) tissues compared to normal tissues (Figures 1F,G).

### Prognostic and Clinicopathological Significance of EXOSC5 in CRC

To investigate whether EXOSC5 expression was correlated with the overall survival and clinicopathological features, IHC staining was performed in 159 human CRC samples, and high EXOSC5 protein expression was detected in 34.0% (54/159) of CRC (Figure 1H). Kaplan-Meier analysis revealed that patients with high EXOSC5 expression had worse overall survival outcomes than patients with low EXOSC5 expression (Figure 1I). Cox multivariate analysis indicated that EXOSC5 expression, along with TNM stage, was an independent risk factor for overall survival of GC patients (Table S2). Furthermore, high EXOSC5 expression positivity correlated with the tumor size (*P* = 0.001) (Table 1). The correlation between EXOSC5 expression and molecular background (KRAS, BRAF, HER2, MLH1, MSH2, MSH6 and PMS2) were analyzed. However, we failed to find potential correlation between EXOSC5 and these parameters (Table S3).

**Table 1:** Correlation between EXOSC5 expression and the clinicopathological parameters of 159 CRC patients.

	**Univariate analysis**				**Multivariate analysis**		
**Clinicopathological parameters**	**Number of cases**	**EXOSC5 expression level**		***P*-value**	**OR**	**95% CI**	***P*-value**
		**High**	**Low**				
Sex				0.722			
Male	106	35	71				
Female	53	19	34				
Age (years)				0.082			
≥65	83	23	60				
<65	76	31	45				
Tumor size				<0.001	1.472	0.805–2.848	0.001
>5 cm	42	25	17				
≤5 cm	117	29	88				
Histologic differentiation				0.331			
Well or moderate	88	27	61				
Poor	71	27	44				
TNM stage				0.022			
I-II	79	20	59				
III-IV	80	34	46				
Serum CEA level				0.061			
>5 ng/ml	55	24	31				
≤5 ng/ml	104	30	74				
Lymphovascular invasion				0.713			
Negative	130	45	85				
Positive	29	9	20				
Perineural invasion				0.475			
Negative	137	48	89				
Positive	22	6	16				

*CEA, carcinoembryonic antigen; TNM, Tumor-Node-Metastasis stage*.

### Knockdown of EXOSC5 Suppressed the Proliferation and Tumorigenesis of CRC Cells

**Figure 2:**
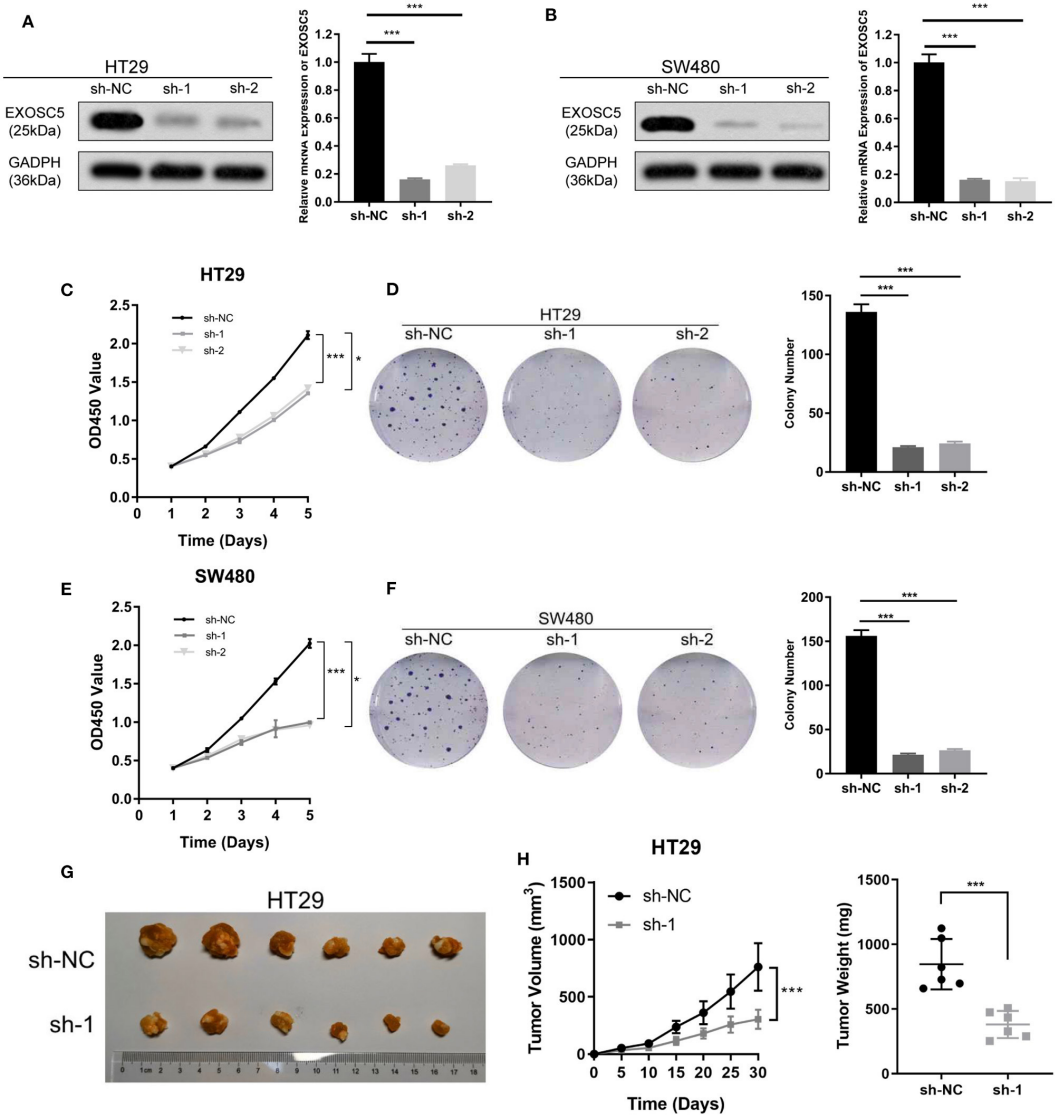
Knockdown of EXOSC5 suppressed the proliferation and tumorigenesis of human CRC cells *in vivo* and *in vitro*. **(A,B)** The efficiency of EXOSC5 knockdown in HT29 and SW480 cells were determined by Western blot, GAPDH was used as a loading control. **(C–F)** Knockdown of EXOSC5 repressed cell proliferation by CCK-8 assays and colony formation assays. **(G)** Tumorigenesis assay by subcutaneous injection of HT29/sh-NC and HT29/sh-EXOSC5 cells in nude mice (*n* = 6/group). **(H)** Tumor volumes were measured by growth curve every 5 days, and weights were measured on the terminal days. The results are presented as the mean ± SD. (*P < 0.05, ***P < 0.001).

EXOSC5 expression in HT29 and SW480 was knocked down by two different shRNA (#1 and 2#). The shRNA suppression efficiency of EXOSC5 expression was confirmed by Western blot and qRT-PCR (Figures 2A,B). Colony formation and CCK-8 assays revealed that knockdown of EXOSC5 significantly repressed HT29 and SW480 cells cell growth compared with control cells (Figures 2C–F). Tumorigenesis assays by subcutaneous injection with stably transfected HT29 cells were performed in nude mice, and tumor growth was monitored. The mean volumes and weights were significantly smaller in the EXOSC5 knockdown tumors than those in the control group (Figures 2G,H).

### Overexpression of EXOSC5 Promoted the Growth of CRC Cells

**Figure 3:**
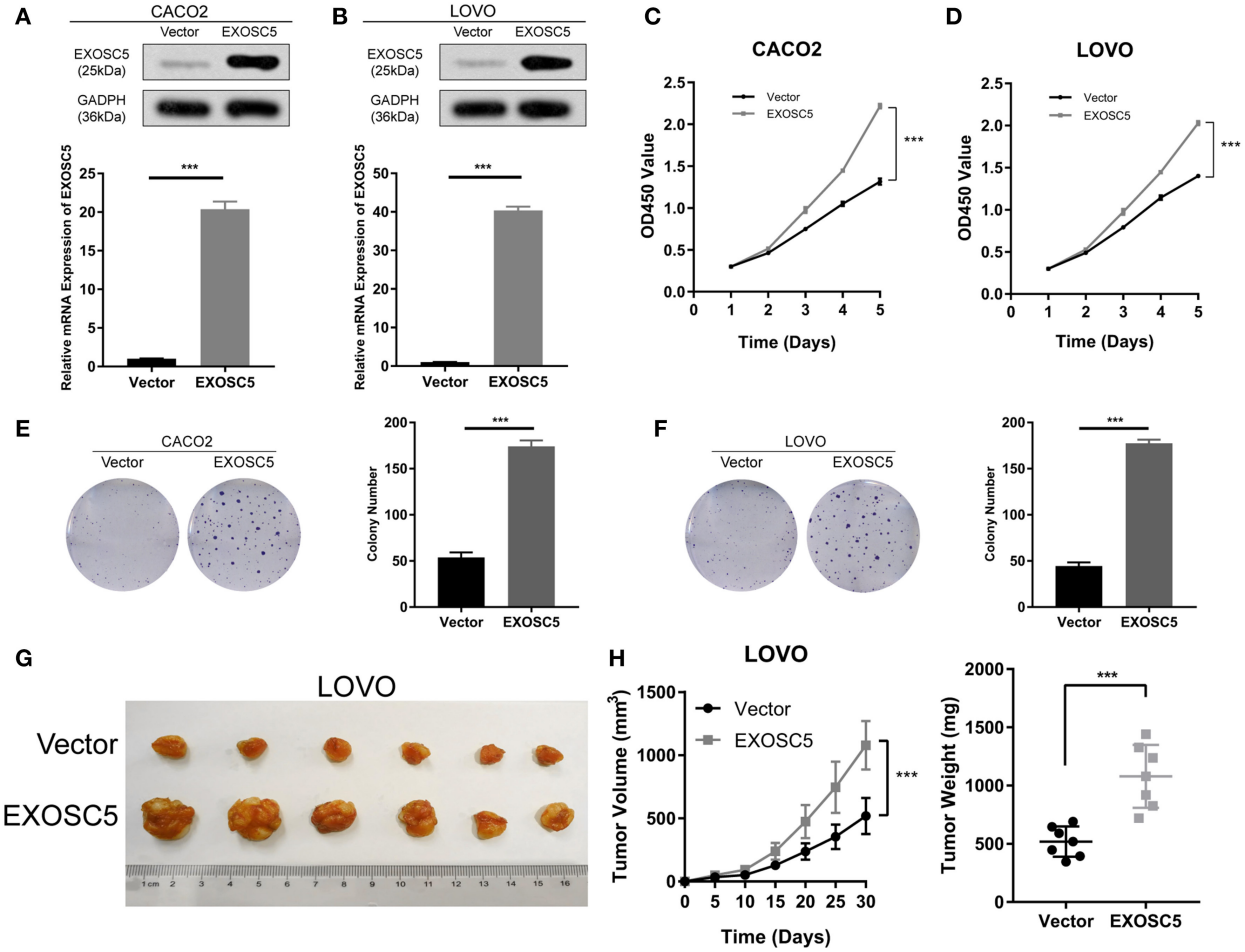
Overexpression of EXOSC5 promoted the proliferation and tumorigenesis of human CRC cells *in vivo* and *in vitro*. **(A,B)** The efficiency of EXOSC5 overexpression in CACO2 and LOVO cells were determined by Western blot, GAPDH was used as a loading control. **(C–F)** Overexpression of EXOSC5 promoted cell proliferation by CCK-8 assays and colony formation assays. **(G)** Tumorigenesis assay by subcutaneous injection of LOVO/Vector and LOVO/EXOSC5 cells in nude mice (*n* = 6/group). **(H)** Tumor volumes were measured by growth curve every 5 days, and weights were measured on the terminal days. The results are presented as the mean ± SD. (***P < 0.001).

The CACO2 and LOVO cell lines of EXOSC5 overexpression were established, the overexpression of EXOSC5 in these cells was confirmed by Western blot and qRT-PCR (Figures 3A,B). The results of CCK-8 and colony formation assays showed that the growth of CACO2 and LOVO cells was promoted by EXOSC5 overexpression compared with negative control cells (Figures 3C–F). Furthermore, to validate these effects *in vivo*, subcutaneous tumorigenesis assays were performed in nude mice. Overexpression of EXOSC5 in the LOVO cells significantly augmented tumor growth *in vivo* (Figures 3G,H).

### EXOSC5 Knockdown Led to G1 Arrest in CRC

**Figure 4:**
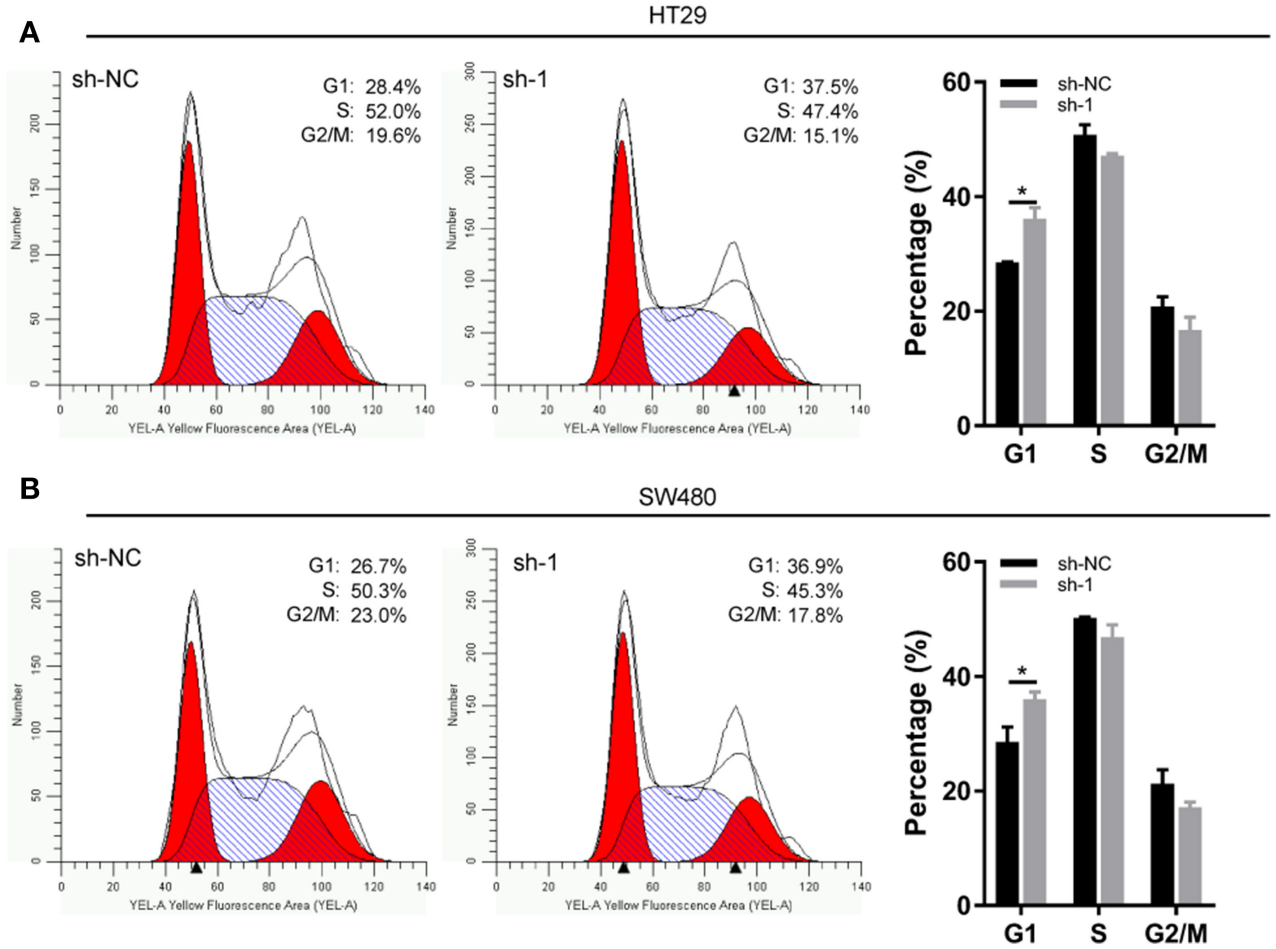
EXOSC5 Knockdown inhibits G1/S transition of CRC cell. **(A,B)** Representative images of the cell cycle assays in HT29 **(A)** and SW480 cells **(B)** after transfection with sh-NC or sh-EXOSC5. Cells were stained with PI and analyzed by flow cytometry. (*p < 0.05).

**Figure 5:**
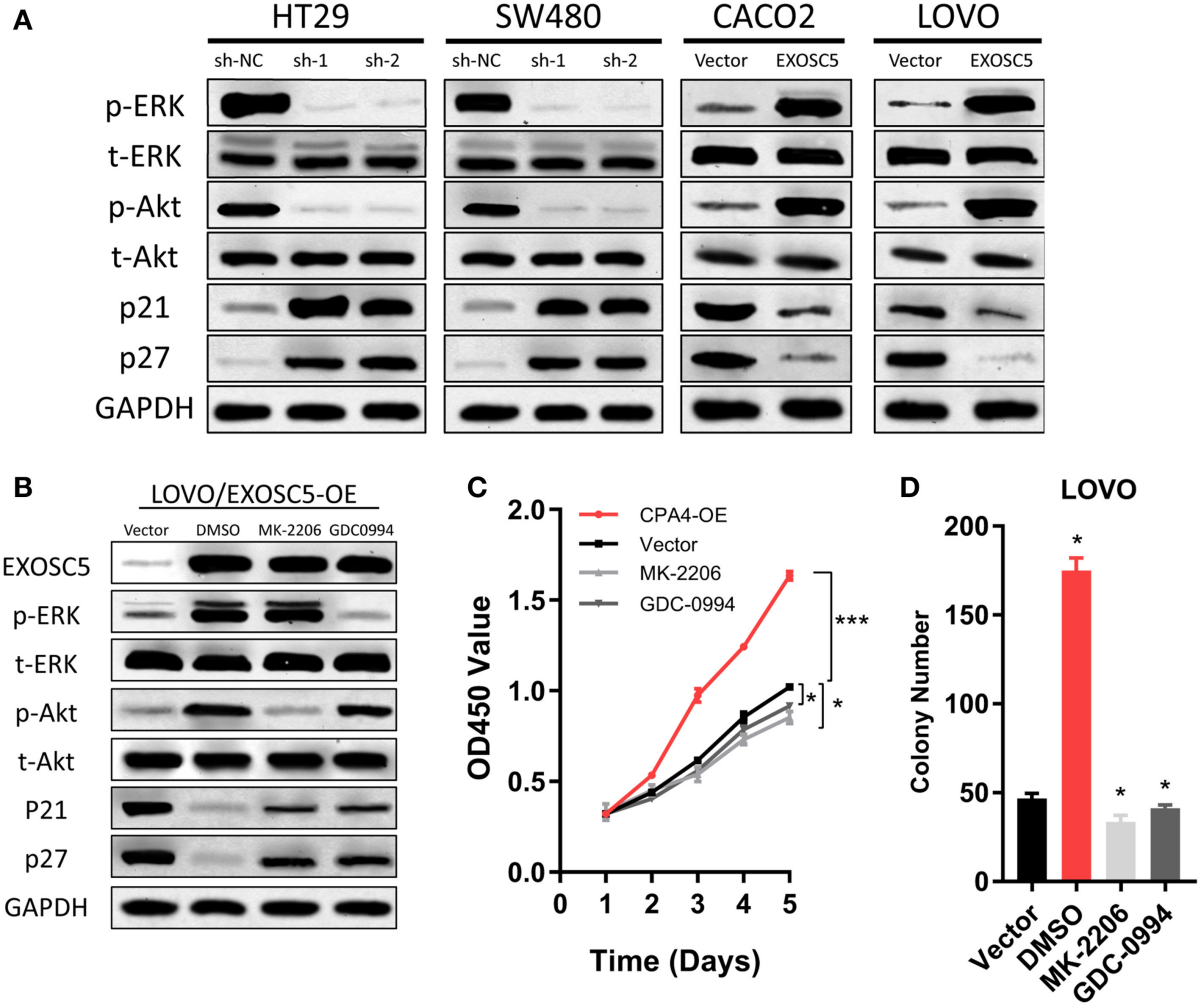
EXOSC5 activated AKT and ERK signaling pathways in CRC cells. **(A)** The levels of phosphorylated ERK, total ERK, phosphorylated Akt, total Akt, cleavages of caspase-9, and caspase-3 were detected in EXOSC5 knockdown and overexpression cells by western blot analysis. GADPH was used as the loading control. **(B)** LOVO cells with EXOSC5 overexpression were treated with the AKT inhibitor MK-2206, the ERK inhibitor U01226 for 24 h. The cells were harvested to measure the expression levels of the indicated proteins by western blot. **(C,D)** Proliferation ability of LOVO cells with EXOSC5 overexpression was determined by CCK-8 **(C)** and colony formation assay **(D)** after treatment with MK-2206, GDC-0994, or DMSO. Error bars represent mean ± SD from 3 independent experiments. (*p < 0.05, ***p < 0.001).

Flow cytometry was conducted to investigate the changes in the cell cycle profile following EXOSC5 knockdown. The percentage of cells in the G1 phase was remarkably augmented for shEXOSC5-transfected HT29 and SW480 cells as compared to that of shNC-transfected cells (*P* < 0.05) (Figures 4A,B). Whereas, there was no significant difference in the percentage of cells in the S or G2/M phases after EXOSC5 knockdown. The data indicated that EXOSC5 knockdown attenuates CRC cell growth by suppressing the G1/S transition. Cyclin-dependent kinases inhibitor proteins p21 and p27, the central regulators of cell cycle transition, were assessed by Western blot. The results showed that p21 and p27 were upregulated in EXOSC5 knockdown cells, while they down-regulated in EXOSC5 overexpression cells compared with those in control cells (Figure 5A).

### EXOSC5 Activates Akt and ERK Pathways in CRC Cells

To find the regulatory mechanism of EXOSC5 in CRC, we measured the phosphorylation and total protein levels of ERK and AKT in EXOSC5 overexpression and knockdown cell lines. Western blot demonstrated that the phosphorylated ERK and AKT decreased in HT29 and SW480 cells after EXOSC5 knockdown, but increased in CACO2 and LOVO cells after overexpression of EXOSC5 compared with negative control cells (Figure 5A).

The EXOSC5 overexpressing LOVO cells were then treated with an AKT inhibitor (MK-2206) or ERK inhibitor (GDC-0994). The result of Western blots showed that Akt and ERK phosphorylation was subsequently inhibited in the EXOSC5 overexpressing LOVO cells (Figure 5B). Moreover, the p21 and p27 expression levels were rescued by the AKT or ERK inhibitors as compared to negative control treated with DMSO (Figure 5B). In addition, CCK-8 and colony formation assays revealed that the growth of these CRC cells promoted by EXOSC5 was reversed after treatment with MK-2206 or GDC-0994 (Figures 5C,D). The above results may show that EXOSC5 plays a role in promoting proliferation of CRC partly by activating the Akt and ERK pathway.

## Discussion

Our study demonstrated for the first time that both the mRNA and protein level of EXOSC5 were upregulated in CRC cell lines and tissue samples. *In vitro* experiments showed that overexpression of EXOSC5 promoted CRC cell proliferation and colony-forming ability. EXOSC5 also promoted tumor growth *in vivo* in mice. Moreover, EXOSC5 knockdown significantly suppressed the cell tumor growth and proliferation both *in vitro* and *in vivo*, and caused G1/S arrest. IHC staining indicated that overexpression of EXOSC5 was associated with worse prognosis, larger tumor size, and advanced tumor stage of CRC patients. Accordingly, EXOSC5 may play an important role in CRC development, and may be a promising biomarker for CRC.

EXOSC5, also known as Rrp46p or CML28, is a non-catalytic component of the RNA exosome complex which has 3′->5′ exoribonuclease activity and participates in a multitude of cellular RNA processing and degradation events (9). EXOSC5 was first identified by Yang et al. (6) and it overexpressed in various epithelial and hematopoietic tumor cell lines, but not in normal tissues (6–8). By using ELISA assay, specific serological responses of EXOSC5 were found in 10–33% of patients with lung cancer, melanoma, and prostate cancer. Given its expression and immunogenicity in a wide variety of malignancies, EXOSC5 merits additional evaluation as a target for antigen-specific immunotherapy (6). Yang et al. found that EXOSC5 forms a homodimer separately from exosome complexes and is either a structural or catalytic component of the machinery that cleaves DNA during apoptosis (10). Wu et al. revealed that EXOSC5 was upregulated in leukemic blasts from patients with acute myelogenous leukemia and chronic myelogenous leukemia blast crisis but is barely detectable in normal bone marrow, normal peripheral blood, or leukemic cells from patients with stable-phase CML (7). Han et al. reported that EXOSC5 was expressed in a large variety of histological tumors, and that EXOSC5 would be of potential use in peptide-based, cancer-specific immunotherapy against a broad spectrum of tumors (11). However, there are limited reports about the functional role and clinical significance of EXOSC5 in solid tumors. As far as we know, this is the first study to report the function and underlying mechanism of EXOSC5 in CRC by *in vivo* and *in vitro* experiments.

We provided the evidences in our study that EXOSC5 knockdown suppressed the phosphorylation of ERK and AKT pathways, and caused the increase in p21 and p27 expression. Flow cytometry also showed that the percentage of G1-phase cells increased significantly in EXOSC5 knockdown cells. Moreover, after treatment with the Akt inhibitor, MK-2206, and ERK inhibitor, GDC-0994, in EXOSC5 overexpressing cells, the cell growth was suppressed, the phosphorylation of Akt, and ERK was significantly decreased, and suppressed p21 and p27 expression were rescued.

According to the above results, we could deduce that EXOSC5 promoting tumor cell growth and proliferation may be partly attributable to its facilitation effect of G1/S transition through ERK and Akt signaling pathways.

Both PI3K/AKT and MAPK/ERK are classic intracellular signaling pathways that are correlated with the regulation of proliferation and cell cycle, and are previously reported to be activated in several kinds of cancer (12, 13). Activation of Akt was shown to overcome cell cycle arrest in G1 phase (14). p21 and p27 are potent cyclin-dependent kinase inhibitors that serve as key regulators of cell cycle at G1 and S phase (15, 16). Increased levels of the p21 and p27 typically cause cells to arrest in the G1 phase of the cell cycle. Both p27 and p21 are members of the “Cip/Kip” family, and share a homologous N-terminal domain, which can bind to CDK complexes. Previous studies have revealed that ERK and AKT signaling were negative regulators of cyclinD1 and CDK inhibitors, including p21 and p27 (17–19). Akt promotes cell-cycle progression through the mechanisms of phosphorylation-dependent 14-3-3 binding to p27Kip1 and cytoplasmic localization (20). ERK and AKT signaling were implicated with the ubiquitylation-dependent proteasomal degradation of p21 and p27 (21). Therefore, we suggested that EXOSC5 has an important role in the tumor progression in CRC through activating the PI3K/AKT and MAPK/ERK signaling pathways. However, the direct downstream targets of EXOSC5 have not been identified. Further studies are warranted to clarify the specific functions of EXOSC5 in CRC patients.

In conclusion, for the first time we have revealed that EXOSC5 was overexpressed in CRC tissues and cell lines. Overexpression of EXOSC5 is significantly associated with tumor size as well as worse oncological outcomes. EXOSC5 promoted CRC cell growth *in vivo* and *in vitro* via activating the ERK and Akt signaling pathways. Our study highlights the oncogenic role and potential mechanism of EXOSC5 in promoting CRC proliferation and tumorigenesis.

## Data Availability

All datasets generated for this study are included in the manuscript and/or the Supplementary Files.

## Ethics Statement

Written informed consent was obtained from all patients and the study was approved by the ethics committees of the BJH.

## Author Contributions

HP and JP designed the study and wrote the draft of the manuscript. LJ revised the draft. LJ and SS conducted the data collection and analysis. HL and ZY prepared the figures and tables the manuscript.

### Conflict of Interest Statement

The authors declare that the research was conducted in the absence of any commercial or financial relationships that could be construed as a potential conflict of interest.

## Supplementary Material

Table S1 IHC score of CRC and normal tissues of 53 patients.

Table S2 Cox multivariate analysis of independent risk factors for overall survival.

Table S3 The correlation between EXOSC5 expression and molecular parameters.
