# Corrigendum: EXOSC5 as a Novel Prognostic Marker Promotes Proliferation of Colorectal Cancer via Activating the ERK and AKT Pathways

**DOI:** 10.3389/fonc.2021.670041

**Published:** 2021-04-09

**Authors:** Hongda Pan, Jingxin Pan, Shibo Song, Lei Ji, Hong Lv, Zhangru Yang

**Affiliations:** ^1^Department of Gastric Surgery, Fudan University Shanghai Cancer Center, Fudan University, Shanghai, China; ^2^Department of Hematology, The Second Affiliated Hospital of Fujian Medical University, Quanzhou, China; ^3^Department of Oncology, Shanghai Medical College, Fudan University, Shanghai, China; ^4^Department of Gastrointestinal Surgery, Beijing Hospital, Beijing, China

**Keywords:** EXOSC5, proliferation, colorectal cancer, Akt signaling pathway, ERK signaling pathway, prognosis

In the original article, there were mistakes in Figure 2 as published. In Figure 2D and **F**, an unintentional error occurred upon using Adobe Illustrator to organize the images, and incorrect images of the colony formation assay for sh-1 and sh-2 in HT29 and SW480 cells were imported by mistake. In Figure 2G, a draft image of tumorigenesis that should have been discarded was imported by mistake. The corrected Figure 2 appears below.

**Figure 2:**
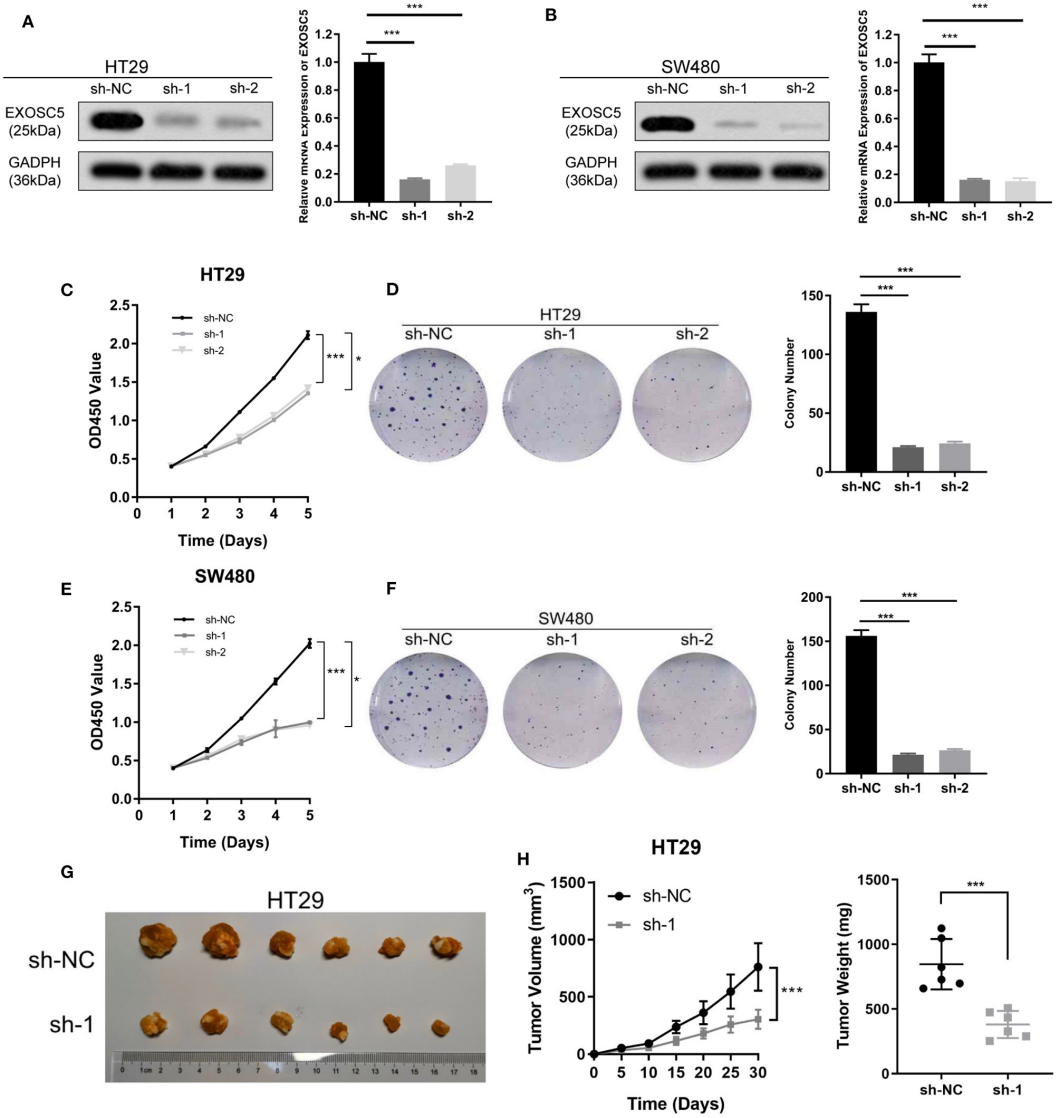
Knockdown of EXOSC5 suppressed the proliferation and tumorigenesis of human CRC cells *in vivo* and *in vitro*. **(A,B)** The efficiency of EXOSC5 knockdown in HT29 and SW480 cells were determined by Western blot, GAPDH was used as a loading control. **(C–F)** Knockdown of EXOSC5 repressed cell proliferation by CCK-8 assays and colony formation assays. **(G)** Tumorigenesis assay by subcutaneous injection of HT29/sh-NC and HT29/sh-EXOSC5 cells in nude mice (*n* = 6/group). **(H)** Tumor volumes were measured by growth curve every 5 days, and weights were measured on the terminal days. The results are presented as the mean ± SD. (*P < 0.05, ***P < 0.001).

The authors apologize for this error and state that this does not change the scientific conclusions of the article in any way. The original article has been updated.

