# Supplementary material for: EXOSC5 as a Novel Prognostic Marker Promotes Proliferation of Colorectal Cancer via Activating the ERK and AKT Pathways
Source: Front Oncol. 2019 Jul 18;9:643. doi: 10.3389/fonc.2019.00643 (PMC6659499; doi:10.3389/fonc.2019.00643)
Supplement: Table S1 — IHC score of CRC and normal tissues of 53 patients. [file Table_1.DOCX]

| Patient No. | Type | Percentage | Intensity | Sum |
| --- | --- | --- | --- | --- |
| 1 | Normal | 1 | 2 | 2 |
| 1 | Cancer | 3 | 1 | 5 |
| 2 | Normal | 1 | 1 | 1 |
| 2 | Cancer | 1 | 3 | 5 |
| 3 | Normal | 1 | 2 | 2 |
| 3 | Cancer | 1 | 2 | 4 |
| 4 | Normal | 0 | 1 | 1 |
| 4 | Cancer | 3 | 1 | 5 |
| 5 | Normal | 1 | 1 | 1 |
| 5 | Cancer | 2 | 1 | 3 |
| 6 | Cancer | 3 | 2 | 6 |
| 6 | Normal | 2 | 0 | 1 |
| 7 | Cancer | 3 | 3 | 6 |
| 7 | Normal | 2 | 1 | 3 |
| 8 | Normal | 2 | 1 | 2 |
| 8 | Cancer | 1 | 1 | 3 |
| 9 | Normal | 1 | 2 | 2 |
| 9 | Cancer | 1 | 0 | 2 |
| 10 | Normal | 1 | 2 | 2 |
| 10 | Cancer | 1 | 1 | 3 |
| 11 | Normal | 0 | 2 | 1 |
| 11 | Cancer | 0 | 2 | 2 |
| 12 | Cancer | 3 | 1 | 5 |
| 12 | Normal | 2 | 3 | 4 |
| 13 | Normal | 1 | 1 | 2 |
| 13 | Cancer | 3 | 0 | 3 |
| 14 | Cancer | 2 | 2 | 5 |
| 14 | Normal | 1 | 1 | 0 |
| 15 | Cancer | 3 | 1 | 5 |
| 15 | Normal | 1 | 1 | 0 |
| 16 | Normal | 1 | 1 | 0 |
| 16 | Cancer | 1 | 1 | 2 |
| 17 | Normal | 1 | 0 | 1 |
| 17 | Cancer | 1 | 1 | 2 |
| 18 | Normal | 0 | 0 | 0 |
| 18 | Cancer | 0 | 1 | 2 |
| 19 | Cancer | 2 | 3 | 4 |
| 19 | Normal | 2 | 1 | 2 |
| 20 | Normal | 2 | 0 | 0 |
| 20 | Cancer | 2 | 2 | 3 |
| 21 | Normal | 1 | 3 | 2 |
| 21 | Cancer | 1 | 2 | 3 |
| 22 | Normal | 1 | 1 | 0 |
| 22 | Cancer | 1 | 0 | 1 |
| 23 | Cancer | 3 | 2 | 4 |
| 23 | Normal | 3 | 2 | 4 |
| 24 | Cancer | 3 | 3 | 6 |
| 24 | Normal | 3 | 2 | 4 |
| 25 | Normal | 2 | 3 | 1 |
| 25 | Cancer | 2 | 1 | 3 |
| 26 | Cancer | 3 | 3 | 6 |
| 26 | Normal | 3 | 1 | 2 |
| 27 | Cancer | 2 | 3 | 4 |
| 27 | Normal | 2 | 2 | 2 |
| 28 | Cancer | 3 | 0 | 3 |
| 28 | Normal | 3 | 0 | 2 |
| 29 | Cancer | 3 | 3 | 6 |
| 29 | Normal | 3 | 1 | 2 |
| 30 | Normal | 0 | 1 | 1 |
| 30 | Cancer | 0 | 1 | 1 |
| 31 | Cancer | 3 | 3 | 6 |
| 31 | Normal | 3 | 2 | 5 |
| 32 | Normal | 1 | 3 | 1 |
| 32 | Cancer | 1 | 1 | 2 |
| 33 | Normal | 0 | 0 | 0 |
| 33 | Cancer | 0 | 2 | 2 |
| 34 | Cancer | 1 | 3 | 3 |
| 34 | Normal | 1 | 0 | 1 |
| 35 | Cancer | 3 | 2 | 4 |
| 35 | Normal | 3 | 1 | 1 |
| 36 | Cancer | 2 | 2 | 3 |
| 36 | Normal | 2 | 1 | 2 |
| 37 | Cancer | 2 | 1 | 3 |
| 37 | Normal | 2 | 2 | 1 |
| 38 | Cancer | 3 | 2 | 4 |
| 38 | Normal | 3 | 1 | 1 |
| 39 | Normal | 1 | 2 | 2 |
| 39 | Cancer | 1 | 1 | 2 |
| 40 | Cancer | 1 | 2 | 3 |
| 40 | Normal | 1 | 2 | 2 |
| 41 | Cancer | 1 | 2 | 3 |
| 41 | Normal | 1 | 1 | 0 |
| 42 | Normal | 1 | 2 | 2 |
| 42 | Cancer | 1 | 1 | 2 |
| 43 | Normal | 2 | 3 | 5 |
| 43 | Cancer | 2 | 0 | 2 |
| 44 | Cancer | 2 | 1 | 3 |
| 44 | Normal | 2 | 3 | 1 |
| 45 | Normal | 1 | 2 | 3 |
| 45 | Cancer | 1 | 0 | 1 |
| 46 | Cancer | 2 | 3 | 4 |
| 46 | Normal | 1 | 2 | 3 |
| 47 | Cancer | 2 | 1 | 3 |
| 47 | Normal | 1 | 1 | 0 |
| 48 | Normal | 1 | 1 | 2 |
| 48 | Cancer | 1 | 1 | 2 |
| 49 | Cancer | 2 | 3 | 4 |
| 49 | Normal | 2 | 0 | 2 |
| 50 | Cancer | 1 | 2 | 3 |
| 50 | Normal | 0 | 1 | 1 |
| 51 | Cancer | 2 | 2 | 3 |
| 51 | Normal | 1 | 0 | 1 |
| 52 | Cancer | 1 | 3 | 3 |
| 52 | Normal | 1 | 3 | 1 |
| 53 | Cancer | 1 | 0 | 1 |
| 53 | Normal | 2 | 1 | 3 |
