# Supplementary material for: EXOSC5 as a Novel Prognostic Marker Promotes Proliferation of Colorectal Cancer via Activating the ERK and AKT Pathways
Source: Front Oncol. 2019 Jul 18;9:643. doi: 10.3389/fonc.2019.00643 (PMC6659499; doi:10.3389/fonc.2019.00643)
Supplement: Table S2 — Cox multivariate analysis of independent risk factors for overall survival. [file Table_2.DOCX]

|  | Univariate analysis | | | Multivariate analysis | | |
| --- | --- | --- | --- | --- | --- | --- |
|  | HR | 95% CI | p value | HR | 95% CI | p value |
| Age（years） |  |  |  |  |  |  |
| < 65 | 1.000 |  |  |  |  |  |
| ≥65 | 0.644 | 0.359-1.056 | 0.220 |  |  |  |
| Sex |  |  |  |  |  |  |
| Male | 1.000 |  |  |  |  |  |
| Female | 0.897 | 0.495-1.485 | 0.791 |  |  |  |
| Tumor size(cm) |  |  |  |  |  |  |
| < 5 | 1.000 |  |  | 1.000 |  |  |
| ≥5 | 1.764 | 1.107-2.853 | 0.013 | 1.243 | 1.054-2.560 | 0.128 |
| Histological grade |  |  |  |  |  |  |
| Well-moderately | 1.000 |  |  | 1.000 |  |  |
| Poorly | 2.462 | 1.353-3.366 | 0.005 | 1.382 | 0.856-2.233 | 0.158 |
| TNM stage |  |  |  |  |  |  |
| 0-II | 1.000 |  |  | 1.000 |  |  |
| III-IV | 2.244 | 1.285-3.250 | 0.003 | 1.787 | 1.134-2.858 | 0.002 |
| EXOSC5 expression |  |  |  |  |  |  |
| Low | 1.000 |  |  | 1.000 |  |  |
| High | 1.818 | 1.014-2.740 | 0.011 | 1.466 | 1.039-2.461 | 0.024 |
| Serum CEA level |  |  |  |  |  |  |
| ≤5 ng/ml | 1.000 |  |  |  |  |  |
| >5 ng/ml | 1.397 | 0.765-1.985 | 0.557 |  |  |  |
| Lymphovascular invasion |  |  |  |  |  |  |
| negative | 1.000 |  |  |  |  |  |
| positive | 1.097 | 0.695-1.284 | 0.991 |  |  |  |
| Perineural invasion |  |  |  |  |  |  |
| negative | 1.000 |  |  |  |  |  |
| positive | 1.197 | 0.565-1.348 | 0.879 |  |  |  |
